# A rare homozygous variant of *MC2R* gene identified in a Chinese family with familial glucocorticoid deficiency type 1: A case report

**DOI:** 10.3389/fendo.2023.1113234

**Published:** 2023-02-24

**Authors:** ShuPing Liu, Ting Zeng, Cheng Luo, DanXia Peng, Xuan Xu, Qin Liu, Qiong Wu, Qin Lu, FuRong Huang

**Affiliations:** ^1^ Department of Children’s Medical Center, Hunan Provincial People’s Hospital, The First Affiliated Hospital of Hunan Normal University, Changsha, Hunan, China; ^2^ Department of Children's Health Care, Liuzhou Maternity and Child Healthcare Hospital, Liuzhou, Guangxi, China; ^3^ Department of Applied and Translational Medicine, GeneMind Biosciences Company Limited, Shenzhen, China

**Keywords:** FGD1, *MC2R*, homozygous mutation, Chinese siblings, ACTH resistance

## Abstract

**Background:**

Melanocortin-2 receptor (*MC2R*), a member of the G protein-coupled receptor family, is selectively activated by adrenocorticotropic hormone (ACTH). variants in *MC2R* are associated with family glucocorticoid deficiency 1 (FGD1).

**Case presentation:**

We first reported a Chinese family with two affected siblings with a homozygotic variant of c.712C>T/p.H238Y in *MC2R*, presenting with skin hyperpigmentation, hyperbilirubinemia, and tall stature. These individuals showed novel clinical features, including congenital heart defects, not been found in other FGD1 patients.

**Conclusions:**

We reported a Chinese family with affected siblings having a homozygotic variant of c.712C>T/p.H238Y in *MC2R*.Our report may expand the genetic and clinical spectrum of FGD1.

## Background

Melanocortin-2 receptor (*MC2R*), also called ACTHR, which is a member of the G protein-coupled receptor family, is selectively activated by adrenocorticotropic hormone (ACTH). Homozygous or compound heterozygous variant in the *MC2R* is the cause of family glucocorticoid deficiency 1 (FGD1) (OMIM # 202200), and the variants cause ACTH resistance in the adrenal cortex. The clinical features include hypoglycemia, seizures, coma, skin hyperpigmentation, hyperbilirubinemia, cholestasis, tall stature, and developmental delays (1–5). Some dysmorphic features, such as a prominent forehead, hypertelorism, a broad nasal bridge, and small, tapering fingers have also been observed (6, 7).

We here reported a Chinese family with two siblings suffering from FGD1. Our findings may broaden the genetic and clinical spectrum of FGD1.

## Case presentation

### Case 1

The 10-year-old boy was referred to our hospital for fever and arrhythmia. The boy was Gestation 1 Parturition 1 (G1P1) and had been delivered by caesarean section after 40 weeks of gestation. His birth weight and height were normal (3900 g, 50 cm), and there was no history of asphyxia or birth trauma. He was born with skin hyperpigmentation. The child was susceptible to infection. An atrial septal defect was found by ultrasonography when he was one year old, and atrioseptopexy was performed. He had intellectual disability and was unable to communicate. The blood glucose levels before admission were unavailable, and whether he had suffered prolonged neonatal hypoglycemia was undetermined. His parents were non-consanguineous without recognizable abnormalities.

**Figure 1:**
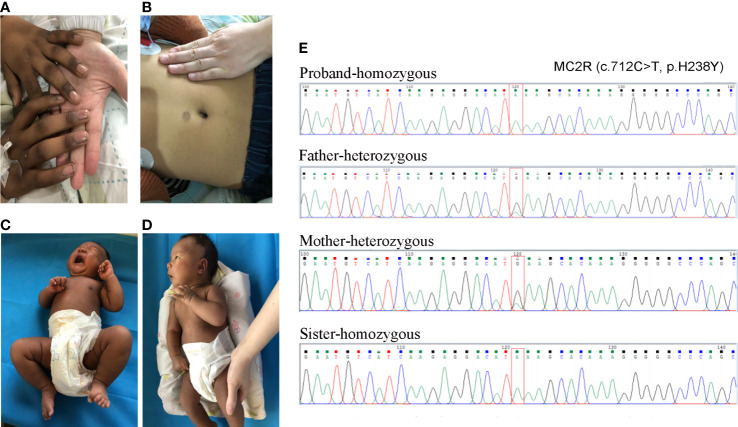
The skin pigmentation and the results of Sanger sequencing. **(A, B)** The skin pigmentation of the brother; **(C, D)** The skin pigmentation of the sister. **(E)** Sanger sequencing results of the variant.

Medical examination: He had a tall stature, height 162 cm (+3.5 SD), weight 48 kg. Skin pigmentation was evident throughout the body, especially the penis and the joints of the fingers and toes (**Figures 1A, B**). His heart rate was 140 beats per minute with arrhythmia. Testicular volume was about 10 ml, and the patient’s penis was 12cm in length and 9 cm in circumference. No armpit hair or public hair was observed. Laboratory examination revealed low plasma cortisol levels, and the plasma ACTH was elevated (**Supplementary Table 1**). Thyroid hormone and androgen levels were low, while The glucose, electrolytes and renin levels remained normal (**Supplementary Table 1**). Based on these laboratory test results, congenital adrenal insufficiency was suspected. After obtaining the consent of the family members, multiplex ligation-dependent probe amplification (MLPA) for congenital adrenocortical hyperplasia (including *CYP21A2*) gene was performed. However, no pathogenic or likely pathogenic mutation was identified. We then performed whole-exome sequencing, and the results showed the proband to be homozygotic for c.712C>T/p.H238Y in *MC2R*. variants come from both parents Sanger sequencing corroborated this.

CNV-seq was performed to identify any possible copy number variations, and no phenotype relevant to CNV was found. Thus, the diagnosis of FGD1 was made according to the clinical phenotypes and genetic testing. Oral hydrocortisone was administered at a dose of 12 mg/m2 body surface area, delivered in three doses. Oral euthyrox was started at a dose of 50 µg/d. After the infection and arrhythmia were treated, he was discharged from the hospital. His serum cortisol levels returned to normal, while ACTH levels remained above normal levels as of one week after discharge.

### Case 2

Fifteen days later, his sister was admitted to our hospital for jaundice. The patient was 27 days old, G4P2, and delivered by caesarean section after 40 weeks and 4 days of gestation. Her Apgar score at 5 min and 10min after birth was 9 and 10. her birth weight was normal (4000 g). She was also born with skin hyperpigmentation (**Figures 1C, D**) but never had hypoglycemia.

**Figure 2:**
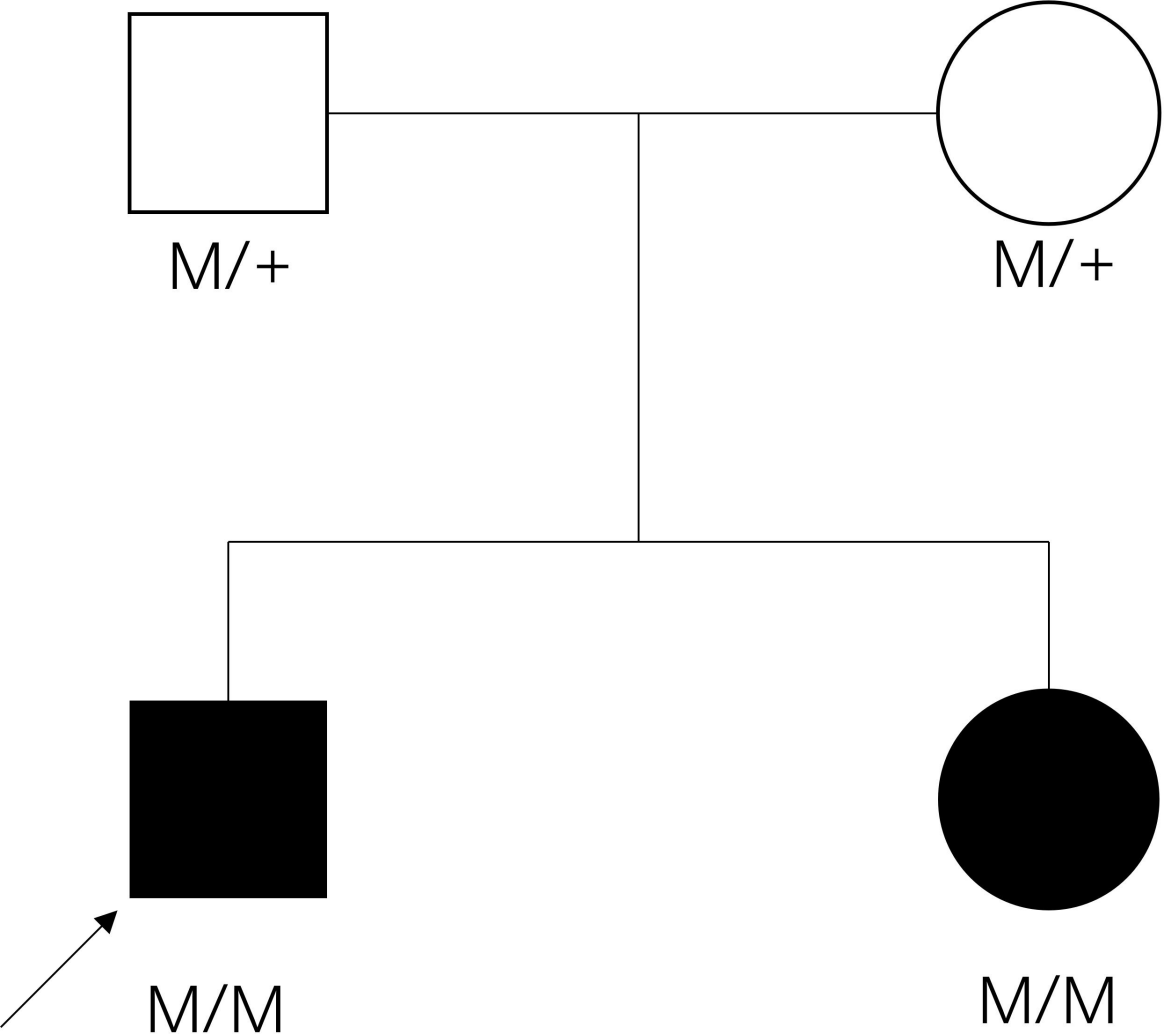
The pedigree of the family. Filled symbol: patient, unfilled symbol: unaffected parents, black arrow: the proband, plus: normal allele, M: mutant allele.

Jaundice appeared four days after birth. It was initially relieved by phototherapy, but gradually worsened after phototherapy was stopped. Laboratory examination revealed that the cortisol level was low, and extremely high morning ACTH was observed (**Supplementary Table 2**). She had normal glucose, electrolytes and renin levels (**Supplementary Table 1**). Thyroid hormone levels were normal, but elevated total bilirubin (Tbil), direct bilirubin (DBIL), indirect bilirubin (IBIL), and total bile acid (TBA) were observed, cardiac ultrasound revealed that she suffered from mild mitral and tricuspid regurgitation, while the electrocardiogram was normal (**Supplementary Table 2**). Sanger sequencing confirmed that the sister had the same homozygotic variants in c.712C>T/p.H238Y in *MC2R* (**Figure 1E**). The pedigree of the family is shown in **Figure 2**. The sister received oral hydrocortisone at a dose of 10 mg/m2 body surface area. To date, no readily visible side effects have been observed. Informed consent was obtained from the parents for the publication of this case.

This variant was located in the transmembrane domain (PM1) and not reported in the dbSNP152, gnomAD, 1000 Genome Database, or Exome Variant Server (PM2). In-silico tools predicted this variant would be pathogenic (SIFT: Damaging, Mutation taster: Disease causing, Polyphen-2: Probably damaging, CADD: Pathogenic and Revel: Damaging), and it was located at a highly conserved site (PP3), the protein model was constructed and polar contacts of wild-type and mutated amino acid residues were compared (**Supplement Figure 1**). A previous work showed it to be a causal variant for FGD1[8], and it was observed in the trans position against other *MC2R* pathogenic variants R145C (PM3). The variant was observed in both affected siblings (PP1). Thus, the variant produced PM1+PM2+PM3+PP1+PP3, which met pathogenic criteria.

## Discussion

ACTH has been shown to activate the *MC2R* to stimulate androgen production in fetal/neonatal mice (8), and deficiency of sex hormones such as 17-alphahydroxyprogesterone, androstenediol, dehydroisoandrosterone, testosterone, progesterone, and dehydroepiandrosterone sulfate (DHAS) had been reported in several FGD1 patients (5, 7, 9–11). Although such patients sometimes had delayed development of pubic hair, other sexual characteristics have not been reported to be affected (10, 12–14). In our case, decreased levels of 17-alpha hydroxyprogesterone and dehydroepiandrosterone were found in the older brother, and slightly lower testosterone level was observed, but he had greater than usual genital size, which may not be associated with deficiency of sex hormones, and we speculated that the excess ACTH leading to the activation of other melanocortin receptors may account for it, while the affected sister showed no such abnormality for receiving timely treatment.

Abnormal thyroid hormone level had been observed in an FGD1 patient as associated features in few cases. Artur Mazur et al. reported hypothyroidism in a Polish patient with familial glucocorticoid deficiency and compound heterozygous p.Leu46fs/p.Val49Met mutation (3). M al Kandari reported that three out of five patients developed hypothyroidism with homozygous frameshift mutation p.Ile154fsX248 (15), and a recent study reported hypothyroidism (TSH 10.61 mIU/L) in a neonatal FGD1 patient with compound heterozygous p.R145C/p.H238Y variant, but the repeated TSH levels without hormone replacement therapy at the age of 1.4 months were normal (16). There is evidence that glucocorticoid inhibits thyrotropin (17), but it could not explain the abnormal thyroid hormone level. The mechanism underlying FGD1-assosiated abnormal thyroid hormone level remains unknown. In our cases, the older brother was found to have abnormal thyroid hormone level, with a low FT3 level and a high TSH level. His FT3 and TSH levels returned to normal after glucocorticoid replacement therapy, but the sister showed no signs of abnormal thyroid hormone level.

A tall stature with normal growth hormone levels is one of the reported characteristics of FGD1 (18). Various melanocortin receptors are expressed in bone cells, and activation of melanocortin receptors can cause increased proliferation and expression of a variety of genes in osteoblastic cells (19). In *MC2R* -/- mice, bone mineral density as well as the thickness of the cortical bone of femur increased (20). Because the FGD1 patients had excess ACTH even after cortisol treatment, activation of other melanocortin receptors in bone cells may account for their tall stature. ACTH had also been found to enhance chondrogenesis in multipotential progenitor cells and matrix production in chondrocytes (21).

Developmental delay and intellectual disability had been described previously in patients with homozygous p.Ile154fsX248 (15). Severe prolonged neonatal hypoglycemia could lead to sequelae such as brain injury, seizure, intellectual disability, cerebral palsy, and visual disturbances (21). In these two cases, the older brother had intellectual disability, but we did not have any data on his blood glucose levels in his neonatal period.

The older brother had congenital atrial septal defect, tricuspid and mitral regurgitation, atrial tachycardia, premature ventricular beats, and QT internal prolongation, while the sister only suffered from tricuspid and mitral regurgitation. To the best of our knowledge, there has been no previous report of congenital heart defects in patients with FGD1. In *MC2R* -/- mice, no morphological abnormalities of the heart were observed, except that the heart rate was attenuated (22). Glucocorticoid receptor (GR) knockout hearts showed aberrant alignment in the compact myocardium with short and disorganized myofibrils (23), indicating a vital role of glucocorticoid signaling in heart physiology and pathophysiology. Whole-exome sequencing and CNV-seq were performed, and no other variant associated with heart disease was found. We could not rule out the possibility of other factors that could account for these defects. Further research is needed to determine the pathogenesis.

The mechanism underlying FGD1 involves unresponsiveness to ACTH due to defective trafficking of the receptor to the cell surface or impaired ligand binding (24). H238Y (located in the TMD6) in our patient was previously reported to be compound heterozygous with R145C, and the patient manifested with hypoglycemia, seizure, skin hyperpigmentation, hyperbilirubinemia, cholestasis, and tall stature (16). A round of *in vitro* site-directed mutagenesis at the same amino acid residue (H238A) resulted in lower expression of *MC2R* on the cell surface, and the binding affinity for ACTH was significantly lower than that for the *hMC2R* WT (25). It has been proposed that H238 may form a second hydrophobic binding pocket with F235 in TM6 of *hMC2R* (25). We speculated that H238Y could attenuate cortisol production by reducing the membrane localization and lowering ACTH binding affinity. Further functional assays were required to reveal the details of the underlying mechanism.

## Conclusions

In this study, we reported two siblings with FGD1 from Hunan Province in China, both harboring homozygous *MC2R* variant, which broaden our understanding of the genetic and clinical spectrum of FGD1.

## Data availability statement

The datasets for this article are not publicly available due to concerns regarding participant/patient anonymity. Requests to access the datasets should be directed to the corresponding author.

## Ethics statement

Written informed consent was obtained from the individual(s), and minor(s)’ legal guardian/next of kin, for the publication of any potentially identifiable images or data included in this article.

## Author contributions

SPL and TZ drafted the initial manuscript, carried out the initial analyses, and reviewed and revised the manuscript. CL, DXP, QLiu, QW, QLu, and FRH designed the data collection instruments, collected data, and critically reviewed the manuscript. XX conceptualized and designed the study, coordinate and supervised data collection, reviewed, and revised the manuscript. All authors contributed to the article and approved the submitted version.

## Supplementary material

Supplementary Figure 1 Protein modulation SWISS-model () was utilized to construct protein model by using the most similar structure (8gy7.1.F, Adrenocorticotropic hormone receptor), and Pymol software () was used to compare the polar contacts of wild-type and mutated amino acid residues.
