# Supplementary figures and images for: A rare homozygous variant of MC2R gene identified in a Chinese family with familial glucocorticoid deficiency type 1: A case report
Source: Front Endocrinol (Lausanne). 2023 Feb 24;14:1113234. doi: 10.3389/fendo.2023.1113234 (PMC10003339; doi:10.3389/fendo.2023.1113234)

## Slide 1
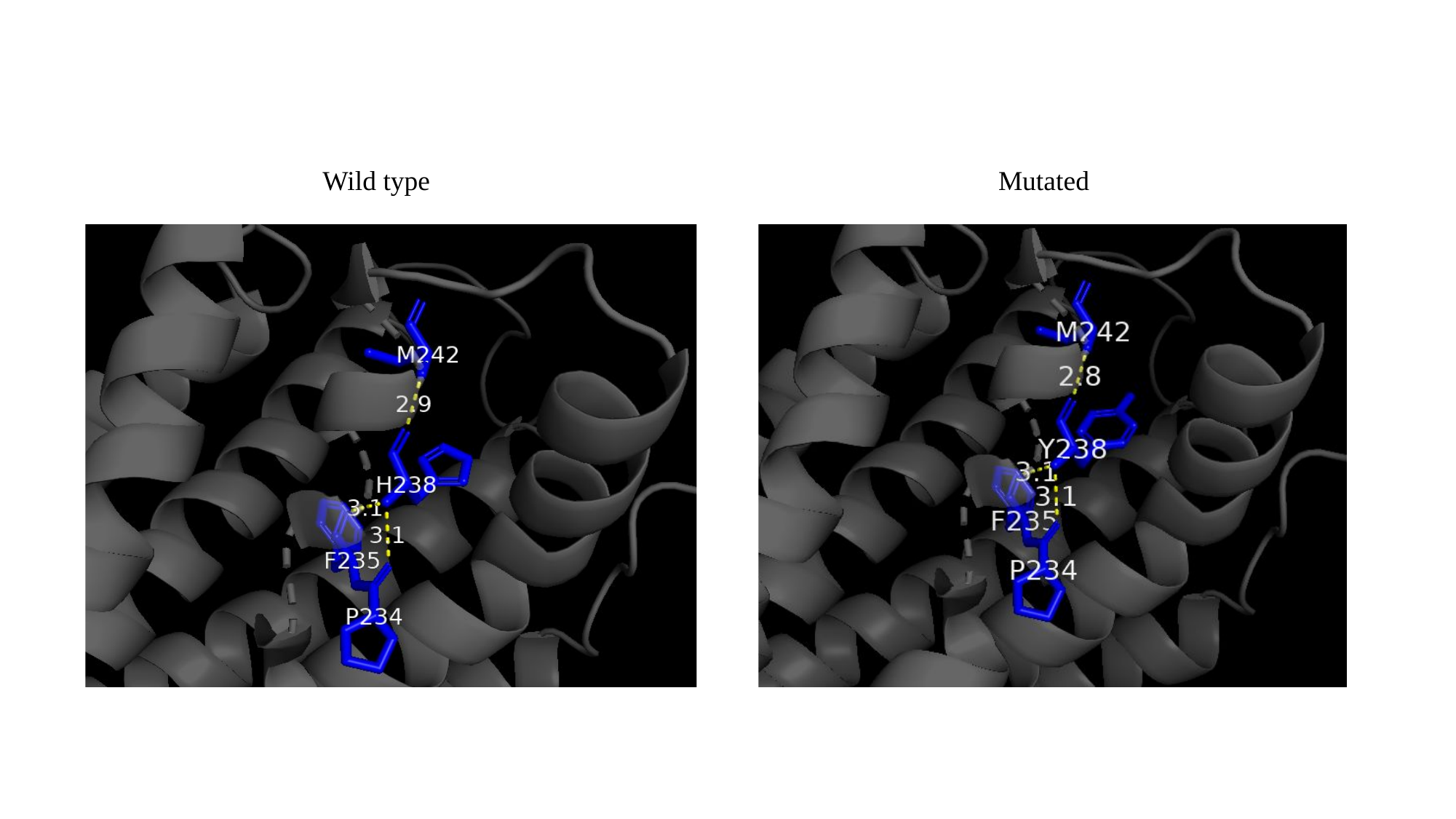

Wild type
Mutated

Supplement: Supplementary Figure 1 — Protein modulation SWISS-model () was utilized to construct protein model by using the most similar structure (8gy7.1.F, Adrenocorticotropic hormone receptor), and Pymol software () was used to compare the polar contacts of wild-type and mutated amino acid residues. [file Presentation_1.pptx]
