# Supplementary material for: A rare homozygous variant of MC2R gene identified in a Chinese family with familial glucocorticoid deficiency type 1: A case report
Source: Front Endocrinol (Lausanne). 2023 Feb 24;14:1113234. doi: 10.3389/fendo.2023.1113234 (PMC10003339; doi:10.3389/fendo.2023.1113234)
Supplement: Supplementary file 2 [file Table_1.docx]

Supplementary Material

A rare homozygous mutation of MC2R gene identified in a Chinese family with familial glucocorticoid deficiency type 1：a case report

Shuping Liu*****, Ting Zeng*****, Cheng Luo，DanXia Peng, Xuan Xu, Qin Liu, Qiong Wu, Qin Lu^3^, FuRong Huang

*** Correspondence:** Xuan Xu 382643958@qq.com

# Supplementary Figures and Tables

**Table 1 Case 1 during hospitalization and after discharge**

|  | Name (reference value) | Hospitalization | Discharge | |
| --- | --- | --- | --- | --- |
|  |  |  | 2 w | 4 w |
| Serum biochemical | ALB (35–55 g/L)  GLB (20–40 g/L)  AST (15–40 U/L)  DBILI (0–6.1 µmol/L)  TBA (0–25 µmol/L)  LDH (100–240 U/L)  BS(3.9-6.1mmol/L)  K+(3.5-5.5mmol/L)  Na+(135-145mmol/L) | 48.7  30.1  23.43  3.5  4.5  201  4.7  5.2  136 | -  -  -  -  -  -  4.6  5.5  139 | 48.7  30.1  23.43  3.5  4.5  -  5.1  4.38  140 |
|  |  |  |  |  |
| Endocrine  and hormone | **8:00 a.m.**  COR (4.5–24 µg/dL)  ACTH (4.7–48.8 pg/ml)  FT3 (3.88–8.02 pmol/l)  FT4 (12.5–21.5 pmol/l)  TSH (0.6–4.84 µIU/ml)  ADS (0–232 pg/ml)  17α-OHP (<2.32 ng/ml)  AD (0.6–3.1 ng/ml)  DHEA (24–537 ng/ml)  T (ng/ml)  IGF1(88–452 µg/l)  Ren(2.8-39.9uIU/ml)  ALDO(0-23.6ng/dL) | 0.328  1005  3.11  12.55  28.67  215  0.74  <0.3  10.5  0.2  263  38  21.5 | 3.627  714  -  -  -  -  0.1  -  -  -  -  -  - | 2.366  374.7  7.06  19.1  10.6  -  -  -  -  -  -  -  - |
|  |  |  |  |  |
| Imaging | B-ultrasound of adrenal glands | Normal | - | - |
|  | B-ultrasound of gonads | Normal | - | - |
|  | Brain and pituitary MRI | Normal | - | - |
|  | Cardiac color Doppler | mild mitral and tricuspid regurgitation | - | - |
|  | Electrocardiograph | ventricular tachycardia, left axis deviation, premature ventricular, QT prolongation | **-** | - |

**Table 2 Case 2 during hospitalization and after discharge**

|  | Name  (reference value) | Hospitalization | Discharge | |
| --- | --- | --- | --- | --- |
|  |  |  | 2 w | 4 w |
| Serum biochemical | ALB (35–55 g/L)  GLB (20–40 g/L)  AST (15–40 U/L)  TBILI (5.1–20 µmol/L)  DBILI (0–6.1 µmol/L)  TBA (0–25 µmol/L)  LDH (100–240 U/L)  BS(3.9-6.1mmol/L)  K+(3.5-5.5mmol/L)  Na+(135-145mmol/L) | 36.6  12.3  4.5  312  24.1  30.9  313  4.2  4.4  135 | 37.3  11.5  42.7  100  15.7  41.9  -  4.7  5.2  135 | -  -  -  -  -  -  -  5.2  4.9  137 |
|  |  |  |  |  |
| Endocrine  and hormone | COR (4.5–24 µg/dL)  ACTH (4.7–48.8 pg/ml)  FT3 (3.0–9.28 pmol/l)  FT4 (11.5–28.3 pmol/l)  TSH (0.77–11 µIU/ml)  ADS (0–232 pg/ml)  17α-OHP (0.82–16.63 ng/ml)  Ren(2.8-39.9uIU/ml)  ALDO(0-23.6ng/dL) | 0.75  1425  5.8  17.4  7.05  273  -  23  3.8 | 0.31  1250  -  -  -  -  -  -  - | 3.72  962.7  6.58  15.61  -  -  -  -  - |
|  |  |  |  |  |
| Imaging | B-ultrasound of adrenal glands | Normal | - | - |
|  | B-ultrasound of gonads | Normal | - | - |
|  | Brain and pituitary MRI | Normal | - | - |
|  | Cardiac color Doppler | Mild mitral and tricuspid regurgitation | - | - |
